# Maximal Oxygen Uptake Is Achieved in Hypoxia but Not Normoxia during an Exhaustive Severe Intensity Run

**DOI:** 10.3389/fphys.2017.00096

**Published:** 2017-02-21

**Authors:** Matthew I. Black, Christopher R. Potter, Jo Corbett, Cain C. T. Clark, Stephen B. Draper

**Affiliations:** ^1^School of Sport, Exercise and Health Sciences, Loughborough UniversityLoughborough, UK; ^2^HE Sport, University Centre, Hartpury CollegeGloucestershire, UK; ^3^Department of Sport and Exercise Science, University of PortsmouthPortsmouth, UK

**Keywords:** VO_2_, VO_2_ kinetics, severe intensity, hypoxia, treadmill running

## Abstract

Highly aerobically trained individuals are unable to achieve maximal oxygen uptake (${\dot{\text{V}}\text{O}}_{2\text{max}}$) during exhaustive running lasting ~2 min, instead ${\dot{\text{V}}\text{O}}_{2}$ plateaus below ${\dot{\text{V}}\text{O}}_{2\text{max}}$ after ~1 min. Hypoxia offers the opportunity to study the (${\dot{\text{V}}\text{O}}_{2}$) response to an exhaustive run relative to a hypoxia induced reduction in ${\dot{\text{V}}\text{O}}_{2\text{max}}$. The aim of this study was to explore whether there is a difference in the percentage of ${\dot{\text{V}}\text{O}}_{2\text{max}}$ achieved (during a 2 min exhaustive run) in normoxia and hypoxia. Fourteen competitive middle distance runners (normoxic ${\dot{\text{V}}\text{O}}_{2\text{max}}$ 67.0 ± 5.2 ml.kg^−1^.min^−1^) completed exhaustive treadmill ramp tests and constant work rate (CWR) tests in normoxia and hypoxia (F_*i*_O_2_ 0.13). The ${\dot{\text{V}}\text{O}}_{2}$ data from the CWR tests were modeled using a single exponential function. End exercise normoxic CWR ${\dot{\text{V}}\text{O}}_{2}$ was less than normoxic ${\dot{\text{V}}\text{O}}_{2\text{max}}$ (86 ± 6% ramp, *P* < 0.001). During the hypoxic CWR test, hypoxic ${\dot{\text{V}}\text{O}}_{2\text{max}}$ was achieved (102 ± 8% ramp, *P* = 0.490). The phase II time constant was greater in hypoxia (12.7 ± 2.8 s) relative to normoxia (10.4 ± 2.6 s) (*P* = 0.029). The results demonstrate that highly aerobically trained individuals cannot achieve ${\dot{\text{V}}\text{O}}_{2\text{max}}$ during exhaustive severe intensity treadmill running in normoxia, but can achieve the lower ${\dot{\text{V}}\text{O}}_{2\text{max}}$ in hypoxia despite a slightly slower ${\dot{\text{V}}\text{O}}_{2}$ response.

## Introduction

Middle distance (800–3000 m) running performance is dependent on the speed that an athlete can sustain for the duration of the event. This speed is dependent on the ability of the locomotor muscles to produce power and resist fatigue (di Prampero et al., 1986; Lacour et al., 1990). The relatively high speed sustained throughout middle distance running events results in an energy demand in excess of the maximal aerobic energy yield (~110–120%), as assessed via pulmonary oxygen uptake (${\dot{\text{V}}\text{O}}_{2}$) and, thus necessitates the integrative contribution from both aerobic and anaerobic pathways (Lacour et al., 1990; Craig and Morgan, 1998; Spencer and Gastin, 2001; Duffield et al., 2005). The 800 m event, for example, requires an ~66 and 34% relative contribution from aerobic and anaerobic metabolism, respectively (Spencer and Gastin, 2001).

The overall energy demand of middle distance running events places these events within the severe, or possibly the extreme intensity domain (Jones and Burnley, 2009). It is assumed that during exercise within the severe or extreme intensity domain, ${\dot{\text{V}}\text{O}}_{2}$ will project exponentially toward the maximal rate of pulmonary oxygen uptake (${\dot{\text{V}}\text{O}}_{2\text{max}}$) until ${\dot{\text{V}}\text{O}}_{2\text{max}}$ is achieved, or exhaustion occurs (Whipp, 1994; Gaesser and Poole, 1996; Poole and Richardson, 1997; Hill and Ferguson, 1999; Jones and Burnley, 2009). However, research utilizing exhaustive constant work rate (CWR) treadmill running of ~2 min and highly aerobically trained middle distance runners (${\dot{\text{V}}\text{O}}_{2\text{max}}$ ≥ 60 ml.kg^−1^.min^−1^) has found that ${\dot{\text{V}}\text{O}}_{2}$ does not achieve ${\dot{\text{V}}\text{O}}_{2\text{max}}$ despite sufficient time for the full response to develop (Draper and Wood, 2005a,b; Sandals et al., 2006; James et al., 2007a,b, 2008). Instead, a submaximal steady state ${\dot{\text{V}}\text{O}}_{2}$ is achieved following ~1 min of exercise with no evidence of a further increase in ${\dot{\text{V}}\text{O}}_{2}$ (Draper and Wood, 2005b).

Previous studies using cross-sectional designs have shown that individuals with a greater ${\dot{\text{V}}\text{O}}_{2\text{max}}$ achieve a lower percentage of ${\dot{\text{V}}\text{O}}_{2\text{max}}$ (${\dot{\text{V}}\text{O}}_{2\text{max}}$) during exhaustive CWR treadmill running of ~2 min (Draper and Wood, 2005a; James et al., 2007a). However, it should be recognized that individuals with a larger ${\dot{\text{V}}\text{O}}_{2\text{max}}$ typically have faster ${\dot{\text{V}}\text{O}}_{2}$ kinetics (Draper and Wood, 2005b; Kilding et al., 2006; Ingham et al., 2007; Marwood et al., 2010). It is therefore unclear why individuals whom possess a large ${\dot{\text{V}}\text{O}}_{2\text{max}}$ and faster ${\dot{\text{V}}\text{O}}_{2}$ kinetics achieve a lower ${\dot{\text{V}}\text{O}}_{2\text{max}}$ than lesser aerobically trained individuals during exercise of this type.

It is well-known that acute hypoxic exposure results in significant reductions in ${\dot{\text{V}}\text{O}}_{2\text{max}}$ relative to values obtained in normoxic conditions (Dill et al., 1966; Dill and Adams, 1971; Engelen et al., 1996; Woorons et al., 2005; Calbet et al., 2015), and the decrement in ${\dot{\text{V}}\text{O}}_{2\text{max}}$ is linearly associated to the fraction of inspired oxygen (FiO_2_) (Lawler et al., 1988). Acute hypoxic exposure, therefore, allows the ${\dot{\text{V}}\text{O}}_{2\text{max}}$ of highly aerobically trained individuals to be artificially and temporarily reduced. Whilst it is recognized that hypoxia may slow ${\dot{\text{V}}\text{O}}_{2}$ kinetics relative to normoxia (Engelen et al., 1996), the magnitude of slowing suggests that ${\dot{\text{V}}\text{O}}_{2}$ kinetics will remain sufficiently fast to permit the manifestation of its full response within <1 min, although evidence from exercise within the severe intensity domain is limited (Heubert et al., 2005). Therefore, hypoxia might provide the opportunity to explore whether highly aerobically trained individuals who are unable to achieve ${\dot{\text{V}}\text{O}}_{2\text{max}}$ during an exhaustive (~2 min) CWR treadmill run in normoxia can achieve a hypoxia reduced ${\dot{\text{V}}\text{O}}_{2\text{max}}$ during a time matched, thus relative intensity matched CWR treadmill run performed in hypoxia.

The purpose of this study, therefore, was to investigate the effect of artificially lowering ${\dot{\text{V}}\text{O}}_{2\text{max}}$ in trained individuals on their ability to attain ${\dot{\text{V}}\text{O}}_{2\text{max}}$ during an exhaustive treadmill run. We hypothesized that highly aerobically trained individuals would be unable to attain ${\dot{\text{V}}\text{O}}_{2\text{max}}$ during a CWR run lasting ~2 min performed in normoxia, but would be able to achieve a hypoxic reduced ${\dot{\text{V}}\text{O}}_{2\text{max}}$.

## Methods

### Subjects

Thirteen males and one female (mean ± SD: age 21 ± 3 y, height 1.76 ± 0.06 m, mass 66.0 ± 7.0 kg) volunteered for the study. All were trained middle distance runners with an 800 m seasonal best of <130 s. Written and informed consent was obtained prior to data collection. Subjects were instructed to report to all testing sessions in a similar state, following their usual pre-competition routine. The study was approved by the institutional ethics committee.

### General procedures

Subjects completed a laboratory familiarization session which was also used to determine appropriate speeds for the CWR tests. The speeds of the CWR tests were adjusted to ensure exhaustion between 105 and 135 s. All tests were performed in an environmental chamber (Sanyo Gallenkamp, PLC, Loughborough), on the same motorized treadmill (ELG 55, Woodway Gmbh, Weil am Rhein, Germany). Air temperature and humidity were controlled at ~16°C and ~40%, respectively. FiO_2_ was manipulated to reflect normoxia (FiO_2_ 0.21) or hypoxia (FiO_2_ 0.13) by a hypoxic unit (Sporting Edge UK Ltd, Sherfield-on-Lodden).

Following familiarization, subjects visited the laboratory on four occasions to a complete ramp incremental tests and CWR tests, in normoxia and hypoxia. The speed of the treadmill was increased by 0.1 km.h^−1^ every 5 s (1.2 km.h^−1^.min^−1^) during the ramp incremental tests, the starting speeds were selected to elicit exhaustion in 8–12 min (Buchfuhrer et al., 1983) in both conditions. The speeds of the CWR tests were based on trial runs completed during the familiarization sessions. If exhaustion was not achieved between 105 and 135 s, the treadmill speed was adjusted and subjects repeated the test on a different day. Trials were randomized to minimize any order effects.

Prior to each CWR run, subjects performed a warm-up on an identical treadmill outside of the environmental chamber. Subjects ran for 5 min at 12 km.h^−1^, 2 min at 15 km.h^−1^, and performed 3 × 10 s runs at the speed of the subsequent CWR test interspersed with 30 s of rest. Following the warm-up the subject entered the environmental chamber. Subjects were encouraged to perform light stretching for 2 min. Following the warm-up and stretching routine, subjects straddled the treadmill for 5 min, allowing the belt to move at the required speed for the test. Heart rate (HR) (recorded every 5 s) and breath-by-breath (${\dot{\text{V}}\text{O}}_{2}$) data were recorded during this period to determine baseline values.

All tests started with the subjects lowering themselves onto the moving treadmill belt. The treadmill was fitted with two handrails, which subjects used to lift themselves onto or clear of the belt. The subject remained in contact with these rails at the start of the test for as long as necessary to reach the required leg speed (typically 2–3 s). The test was stopped when subjects were unable to continue and lifted themselves clear of the treadmill belt.

### Data acquisition

Throughout testing, subjects wore a chest strap and HR was measured using short-range telemetry (810i; Polar Electro Oy, Kempele, Finland), and breathed through a low-dead space (90 ml), low resistance (5.5 cm H_2_O at 510 L.min^−1^) mouthpiece and turbine assembly. Gases were collected continuously from the mouthpiece through a 2 m sampling line (0.5 mm internal diameter) to a quadrupole mass spectrometer (MSX 671: Ferraris Respiratory Europe Ltd, Hertford, UK) where they were analyzed for O_2_, CO_2_, Ar and N_2_. Expired volumes were determined using a turbine volume transducer (Interface Associates, Alifovieja, US). The mass spectrometer and turbine were calibrated before each test using mixtures of known composition (Linde Gas, London, UK), and a 3 L calibration syringe (Hans Rundolf, KS), respectively. Two identical quadrupole mass spectrometers were used; one was placed outside the environmental chamber to accurately determine the internal environmental conditions, this system was calibrated against outside atmospheric air (20.94% O_2_, 0.04% CO_2_, 0.93% Argon, and 78.08% N_2_) and a normoxic gas bottle (14.99% O_2_, 5.01% CO_2_, 5.02% Argon, and 74.98% N_2_). The second system was placed inside the environmental chamber and was calibrated against the environmental conditions provided by the other mass spectrometer and a gas bottle of known composition; the normoxic gas bottle was used during normoxic testing, and a gas bottle composed of 5% O_2_, 5.01% CO_2_, 5.02% Argon, and 84.97% N_2_ was used in hypoxia. The volume and concentration signals were time aligned, accounting for transit delay in capillary gas and analyser rise time relative to the volume signal. ${\dot{\text{V}}\text{O}}_{2}$, ${\dot{\text{V}}\text{CO}}_{2}$, ${\dot{\text{V}}}_{E}$ were calculated for each breath.

### Data analysis

Moving 15 s averages were used to calculate ${\dot{\text{V}}\text{O}}_{2}$, ${\dot{\text{V}}\text{CO}}_{2}$, and ${\dot{\text{V}}}_{E}$ for every complete 15 s period throughout all tests. ${\dot{\text{V}}\text{O}}_{2\text{max}}$ was defined as the highest 15 s ${\dot{\text{V}}\text{O}}_{2}$ value attained during the ramp incremental tests, and ${\dot{\text{V}}\text{O}}_{2\text{peak}}$ was the highest 15 s ${\dot{\text{V}}\text{O}}_{2}$ value achieved during the CWR tests. HR was recorded every 5 s and the highest value achieved during the ramp incremental test was taken as maximum HR (HR_max_) and the highest value recorded during the CWR exercise was the peak HR (HR_peak_).

The breath-by-breath ${\dot{\text{V}}\text{O}}_{2}$ data from the CWR tests were initially examined to exclude errant breaths caused by coughing, swallowing, etc., and values lying more than 4 SD from the local mean were removed. Subsequently, the breath-by-breath data were converted to second-by-second data using linear interpolation and time aligned to the start of the test. The first 15 s of data were removed to account for the cardio-dynamic phase (Murias et al., 2011). A single exponential model was used to characterize ${\dot{\text{V}}\text{O}}_{2}$ kinetics as described in the following equation:

(1) $$\dot{\text{V}}{\text{O}}_{2}\left(t\right)=\dot{\text{V}}{\text{O}}_{2}\;\text{baseline}+A\left(1-\left({\text{e}}^{-\left(t-\delta \right)/\tau }\right)\right)$$

where ${\dot{\text{V}}\text{O}}_{2}$ (*t*) represents the absolute ${\dot{\text{V}}\text{O}}_{2}$ at a given time (*t*), ${\dot{\text{V}}\text{O}}_{2}$ baseline is the average of the ${\dot{\text{V}}\text{O}}_{2}$ measured over the final 120 s of quiet standing, *A* is the asymptotic amplitude, τ is the time constant of the exponential response and δ is a delay. No parameters were constrained.

### Statistical analysis

Data were tested for normality (Duffy and Jacobsen, 2001) and was found to be normally distributed. Two-way (test × condition) repeated measures ANOVA was employed to determine the effect of hypoxia on ${\dot{\text{V}}\text{O}}_{2}$, minute ventilation (${\dot{\text{V}}}_{E}$), ventilatory equivalents (i.e., ${\dot{\text{V}}}_{E}$/ ${\dot{\text{V}}\text{O}}_{2}$, ${\dot{\text{V}}}_{E}$/${\dot{\text{V}}\text{CO}}_{2}$) and HR. *Post hoc t-*tests with Bonferroni correction were used to explore the origin of any significant interaction effect. Paired *t-*tests were used to explore differences in estimates of the modeled ${\dot{\text{V}}\text{O}}_{2}$ data in normoxia and hypoxia. Pearson's Product Moment Correlation was used to investigate the relationship between ${\dot{\text{V}}\text{O}}_{2\text{max}}$, CWR running speed, and the % ${\dot{\text{V}}\text{O}}_{2\text{max}}$ achieved during the CWR tests. The relationship between the difference in running speed and the difference in % ${\dot{\text{V}}\text{O}}_{2\text{max}}$ achieved during the normoxic and hypoxic CWR tests was also investigated. Statistical significance was set at *P* < 0.05. Data are presented as mean ± SD unless otherwise stated.

## Results

The ${\dot{\text{V}}\text{O}}_{2\text{max}}$ measured in the normoxic ramp incremental test was 4.40 ± 0.42 L.min^−1^ (67.0 ± 5.2 ml.kg^−1^.min^−1^) and HR_max_ was 185 ± 7 bpm. Hypoxia reduced ${\dot{\text{V}}\text{O}}_{2\text{max}}$ to 2.97 ± 0.27 L.min^−1^ (45.1 ± 3.0 ml.kg^−1^.min^−1^; *P* < 0.001) and HR_max_ to 181 ± 6 bpm; *P* < 0.05).

**Figure 1:**
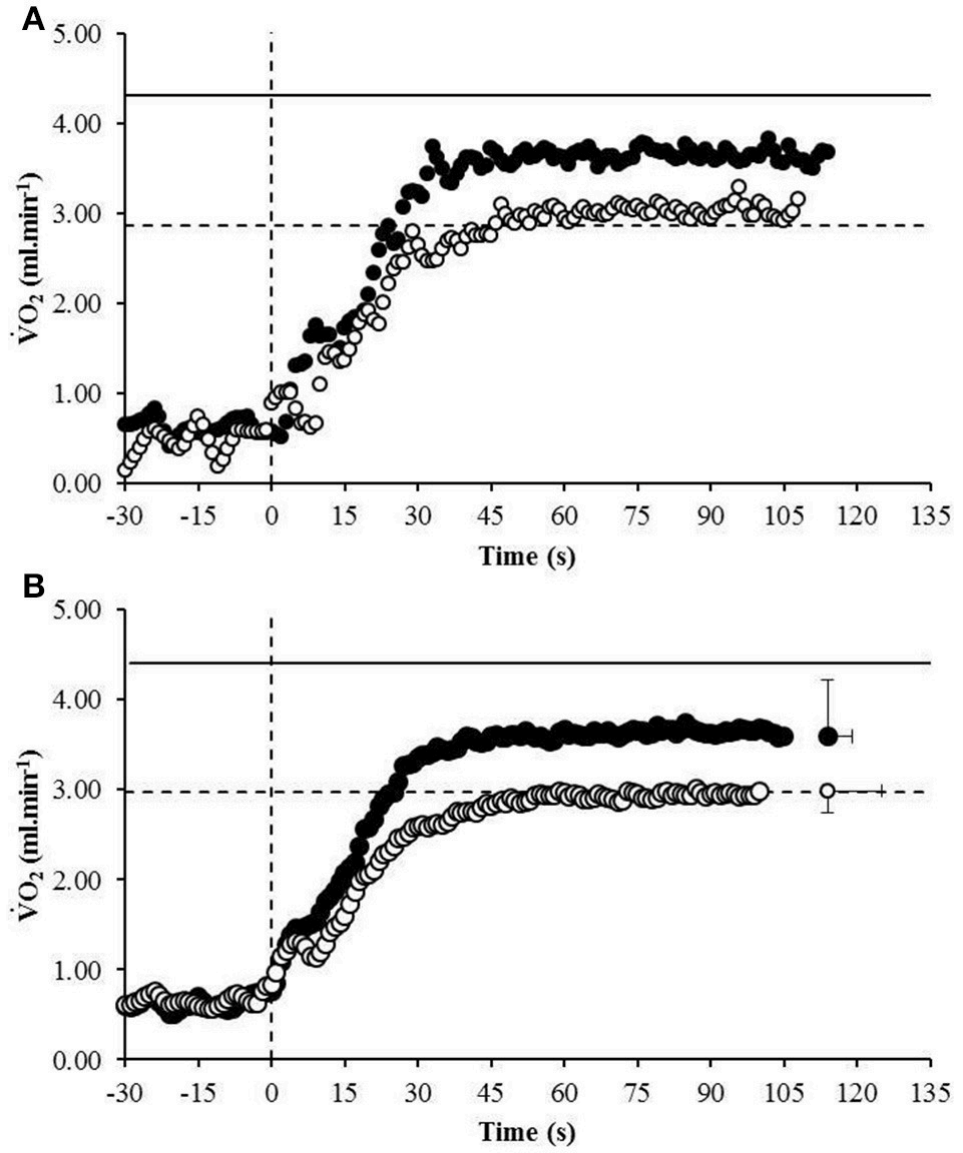
**The ${\dot{\text{V}}\text{O}}_{2}$ response of a representative participant (A)** and the group mean **(B)** to the normoxic CWR test (black circles) and hypoxic CWR test (white circles). The ${\dot{\text{V}}\text{O}}_{2\text{max}}$ to the normoxic ramp (solid line) and hypoxic ramp (broken line) is also provided. Error bars represent the standard error of measurement. For clarity the error bars are omitted for all but the final data point.

**Figure 2:**
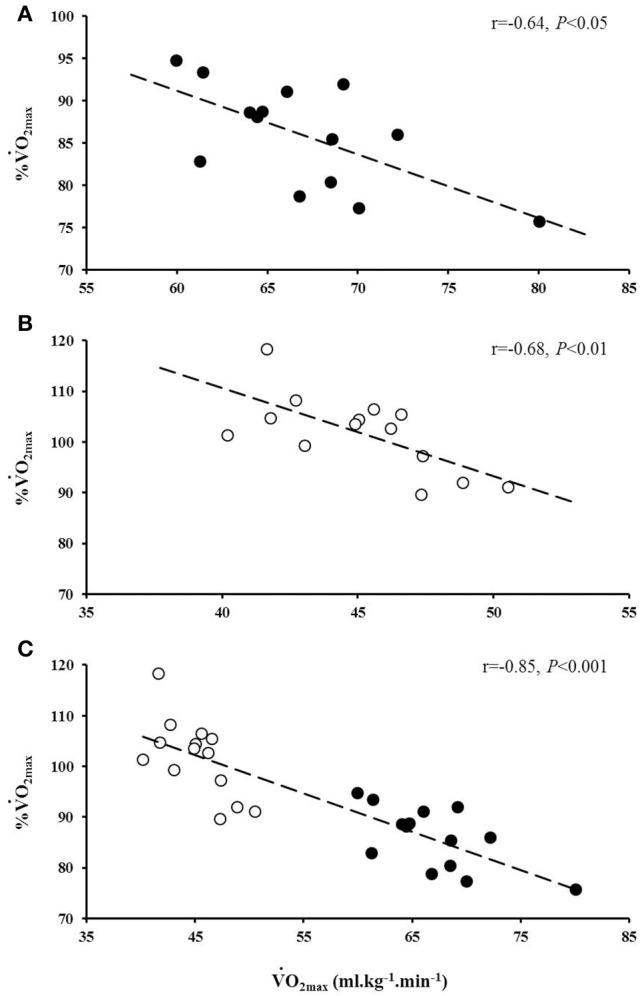
**The relationship between ${\dot{\text{V}}\text{O}}_{2\text{max}}$ and the percentage ${\dot{\text{V}}\text{O}}_{2\text{max}}$ (%${\dot{\text{V}}\text{O}}_{2\text{max}}$) achieved during the normoxic (A**, black circles) and hypoxic (**B**, white circles) CWR tests. The combined relationship between ${\dot{\text{V}}\text{O}}_{2\text{max}}$ and the % ${\dot{\text{V}}\text{O}}_{2\text{max}}$ achieved in normoxia and hypoxia is also provided **(C)**.

The average speed utilized for the normoxic CWR trials was 22.0 ± 1.0 km.h^−1^ which resulted in a trial duration of 114 ± 11 s (range: 100 s to 130 s). The speed of the hypoxic CWR trial was performed at a significantly slower speed (20.5 ± 1.0 km.h^−1^; *P* < 0.001) to ensure a similar duration of trial between conditions. The duration of the hypoxic CWR trial (114 ± 11 s, range: 105 s to 135 s) was not significantly different to the duration of the normoxic CWR trial (114 ± 5 s, range: 105 s to 125 s) (*P* > 0.05). Normoxic ${\dot{\text{V}}\text{O}}_{2\text{max}}$ was not achieved during the normoxic CWR trial (3.79 ± 0.47 L.min^−1^; 86 ± 6% ${\dot{\text{V}}\text{O}}_{2\text{max}}$; *P* < 0.05; Figure 1).However, subjects attained hypoxic ${\dot{\text{V}}\text{O}}_{2\text{max}}$ during the hypoxic CWR trial (3.02 ± 0.30 L.min^−1^; 102 ± 8%; *P* > 0.05; Figure 1). ${\dot{\text{V}}\text{O}}_{2\text{max}}$ was inversely associated with ${\dot{\text{V}}\text{O}}_{2\text{max}}$ achieved during the normoxic (*r* = −0.64, *P* < 0.05) and hypoxic (*r* = −0.68, *P* < 0.01) CWR trials, and when the normoxic and hypoxic trials were combined (*r* = −0.85, *P* < 0.001; Figure 2). Condition-specific HR_max_ was attained during normoxic (189 ± 7 bpm) and hypoxic (181 ± 7 bpm) CWR trials (*P* > 0.05). The parameters of the modeled ${\dot{\text{V}}\text{O}}_{2}$ data are presented in Table 1. No relationships were observed between speed and % ${\dot{\text{V}}\text{O}}_{2}$ achieved during the CWR trials performed in normoxia (*r* = 0.34, *P* > 0.05), hypoxia (*r* = −0.16, *P* > 0.05), or the difference in speed and ${\dot{\text{V}}\text{O}}_{2}$ between the normoxic and hypoxic CWR trials (*r* = −0.05, *P* > 0.05).

**Table 1:** **The parameters of the modeled ${\dot{\text{V}}\text{O}}_{2}$ response to CWR exercise in normoxia and hypoxia**.

	**Normoxia**	**Hypoxia**	***P-*****value**
Ramp ${\dot{\text{V}}\text{O}}_{2\text{max}}$ (L.min^−1^)	4.40 ± 0.42	2.97 ± 0.27	<0.001
CWR ${\dot{\text{V}}\text{O}}_{2\text{peak}}$ (L.min^−1^)	3.79 ± 0.47	3.02 ± 0.30	<0.001
Baseline O_2_ (L.min^−1^)	0.60 ± 0.11	0.67 ± 0.15	>0.05
A (L.min^−1^)	2.45 ± 0.50	1.61 ± 0.27	<0.001
Baseline + A (L.min^−1^)	3.05 ± 0.51	2.28 ± 0.21	<0.001
τ (s)	10.4 ± 2.6	12.7 ± 2.8	<0.05
δ (s)	7.6 ± 2.6	7.4 ± 3.3	>0.05

No significant interaction effect was observed for ${\dot{\text{V}}}_{E}$ (*P* > 0.05) with no significant main effect for condition (normoxia, 137.3 ± 17.5 L.min^−1^; hypoxia 130 ± 16.2 L.min^−1^; *P* > 0.05), but a significant main effect for test (ramp, 128.0 ± 16.6 L.min^−1^; CWR, 139.2 ± 16.0 L.min^−1^; *P* < 0.001). There was a significant interaction effect for ${\dot{\text{V}}}_{E}$/${\dot{\text{V}}\text{O}}_{2}$ (*P* < 0.05) with significant main effects for condition (normoxia, 35.3 ± 6.7 L.min^−1^; hypoxia 43.7 ± 4.7 L.min^−1^; *P* < 0.001) and test (ramp, 128.0 ± 16.6 L.min^−1^; CWR, 139.2 ± 16.0 L.min^−1^; *P* < 0.001). There was a significant interaction effect for ${\dot{\text{V}}}_{E}$/${\dot{\text{V}}\text{CO}}_{2}$ (*P* < 0.01) with significant main effects for condition (normoxia, 28.2 ± 3.7 L.min^−1^; hypoxia 31.6 ± 6.0 L.min^−1^; *P* < 0.05), but no significant difference for test (ramp, 29.8 ± 5.0 L.min^−1^; CWR, 26.2 ± 3.4 L.min^−1^; *P* > 0.05).

## Discussion

The principle novel finding of the current study was that despite being unable to attain ${\dot{\text{V}}\text{O}}_{2\text{max}}$ during normoxic CWR running lasting ~2 min, highly aerobically trained individuals could achieve a hypoxia reduced ${\dot{\text{V}}\text{O}}_{2\text{max}}$ during CWR running of a matched duration, thus of a similar relative intensity. This is the first study to demonstrate that subjects whose ${\dot{\text{V}}\text{O}}_{2}$ plateaued below ${\dot{\text{V}}\text{O}}_{2\text{max}}$ during an exhaustive CWR run, were subsequently able to attain ${\dot{\text{V}}\text{O}}_{2\text{max}}$ when the exercise bout was replicated in hypoxic conditions despite a slowed ${\dot{\text{V}}\text{O}}_{2}$ response.

Previous research has demonstrated that during normoxic CWR running lasting ~2 min, more highly aerobically trained individuals achieved a lower ${\dot{\text{V}}\text{O}}_{2\text{max}}$ (Draper and Wood, 2005a; James et al., 2007a). In agreement with these findings, the current study reported an inverse association between ${\dot{\text{V}}\text{O}}_{2\text{max}}$ and the ${\dot{\text{V}}\text{O}}_{2\text{max}}$ achieved during the normoxic CWR trial (Figure 2). To gain further insight into the relationship between ${\dot{\text{V}}\text{O}}_{2\text{max}}$ and ${\dot{\text{V}}\text{O}}_{2\text{max}}$ achieved the present study investigated whether a hypoxia induced reduction in ${\dot{\text{V}}\text{O}}_{2\text{max}}$ may permit highly aerobically trained individuals to attain ${\dot{\text{V}}\text{O}}_{2\text{max}}$ during exhaustive CWR running at a matched relative intensity. The acute hypoxic exposure reduced ${\dot{\text{V}}\text{O}}_{2\text{max}}$ by ~32%, consistent with previous reports (Engelen et al., 1996; Martin and O'Kroy, 1993; Woorons et al., 2005), and subject to this reduction ${\dot{\text{V}}\text{O}}_{2\text{max}}$ was achieved (Figure 1B). No relationship was observed between % ${\dot{\text{V}}\text{O}}_{2\text{max}}$ achieved and the running speed during the CWR tests in normoxia or hypoxia, nor the difference in speed between conditions (i.e., normoxia and hypoxia) and the difference in %VO_2max_ achieved (all *P* > 0.05), suggesting that ${\dot{\text{V}}\text{O}}_{2\text{max}}$ may be an important parameter in determining whether an individual may be able to achieve their ${\dot{\text{V}}\text{O}}_{2\text{max}}$ during this type of exercise. Furthermore, these findings highlight that further improvements in ${\dot{\text{V}}\text{O}}_{2\text{max}}$ are of less benefit to high-intensity exercise performance compared to similar gains in anaerobic capability. These findings perhaps seem incongruous with the high ${\dot{\text{V}}\text{O}}_{2\text{max}}$ values typically reported in elite 800 m runners (Svedenhag and Sjödin, 1984; Ingham et al., 2008) that they are apparently unable to fully utilize. However, such a high ${\dot{\text{V}}\text{O}}_{2\text{max}}$ value may be due to the high volume of interval training performed by these athletes (Helgerud et al., 2007). There have certainly been instances where performance at altitude would indicate that the decrement in ${\dot{\text{V}}\text{O}}_{2\text{max}}$ may not substantially impair performance. For example, Ralph Doubell equalled the World Record at the 1968 Mexico Olympics which was performed at an altitude of 2,240 m above sea level; a feat that would seem implausible if one's ${\dot{\text{V}}\text{O}}_{2\text{max}}$ was a necessity for optimum performance.

The ${\dot{\text{V}}\text{O}}_{2}$ kinetics were similar to values previously reported during investigations utilizing similarly highly aerobically trained runners during CWR running lasting ~2 min (Draper and Wood, 2005a,b; Draper et al., 2008). The phase I time delays are also similar to those reported by Wilkerson et al. (2004). Consistent with Engelen et al. (1996), we found a slower phase II τ in the hypoxic condition (Table 1). However, it should be noted that despite a slower phase II τ, resulting in ~10 s difference in the attainment of the ${\dot{\text{V}}\text{O}}_{2}$ amplitude, hypoxic ${\dot{\text{V}}\text{O}}_{2\text{max}}$ was achieved. Conversely, despite faster ${\dot{\text{V}}\text{O}}_{2}$ kinetics normoxic ${\dot{\text{V}}\text{O}}_{2\text{max}}$ was not attained. Instead, there was an evident ${\dot{\text{V}}\text{O}}_{2}$ plateau in normoxia at ~86% normoxic ${\dot{\text{V}}\text{O}}_{2\text{max}}$. The occurrence of a ${\dot{\text{V}}\text{O}}_{2}$ plateau, rather than a continued trajectory toward ${\dot{\text{V}}\text{O}}_{2\text{max}}$ and indeed the energy demands of the exercise, questions contemporary models of ${\dot{\text{V}}\text{O}}_{2}$ kinetics during CWR exercise lasting ~2 min in this highly aerobically trained group. Interestingly, this same response is not evident during exhaustive cycle ergometry of a similar duration whereby ${\dot{\text{V}}\text{O}}_{2}$ continues to increase throughout, although maximum values are not attained (Draper et al., 2003). At present the reasons for the differences between exercise modes in ${\dot{\text{V}}\text{O}}_{2}$ response to severe intensity exercise are unclear. Increasing oxygen uptake has been associated with reduced efficiency arising from factors, such as metabolite accumulation, limitations in substrate availability, pH disturbance, increased muscle temperature, and altered motor unit recruitment (Grassi et al., 2015). Indeed, it is well established that the patterns of muscle action, including the relative proportion of eccentric and concentric contraction and the contribution of the stretch-shortening cycle differ between running and cycling (van Ingen-Schenau et al., 1997; Bijker et al., 2002). These effects might, at least in part, contribute to the between mode differences in ${\dot{\text{V}}\text{O}}_{2}$ kinetics, particularly for higher work rates where the use of elastic energy is optimized (Dalleau et al., 1998); whether or not increased stored energy during the stretch shortening cycle can help maintain efficiency despite increased metabolic fatigue warrants further investigation.

Consistent with previous investigations, we found that HR_max_ was greater in normoxia than hypoxia (Benoit et al., 1995; Mollard et al., 2007). However, similar to our ${\dot{\text{V}}\text{O}}_{2}$ findings normoxic HR_max_ was not achieved during the normoxic CWR test (Draper and Wood, 2005a,b), but hypoxic HR_max_ could be achieved during the hypoxic CWR trial. Assuming HR_max_ is needed to achieve maximal cardiac output (Q_max_), these findings suggest that Q_max_ was not achieved during the normoxic CWR test. Despite a lower HR_max_ in hypoxia relative to normoxia, previous findings have shown that hypoxia has no effect on Q_max_ (Mollard et al., 2007), implying a compensatory increase in maximal stroke volume in hypoxia. Therefore, the inability to achieve ${\dot{\text{V}}\text{O}}_{2\text{max}}$ in the normoxic CWR trial may be associated with submaximal cardiac output. However, further investigation that assesses cardiac output and blood flow is necessary to gain insight into Q_max_ as a potential limiting factor in the attainment of ${\dot{\text{V}}\text{O}}_{2\text{max}}$ during this type of exercise.

Although end exercise ${\dot{\text{V}}}_{E}$ was greater during the CWR trials relative to the ramp incremental tests, this was not different between normoxia and hypoxia. Furthermore, we observed no differences in ventilatory equivalents between conditions (i.e., normoxia and hypoxia). These similar ventilatory responses might serve to attenuate or prevent the exercise induced arterial hypoxemia that has been described in highly aerobically trained individuals (Dempsey et al., 1984; Powers et al., 1988, 1992; Caillaud et al., 1993; review Prefaut et al., 2000). In normoxia, the increased ${\dot{\text{V}}}_{E}$ during CWR exercise would likely increase the work of breathing thereby compromising limb muscle blood flow (Wetter et al., 1999). In hypoxia, the PO_2_ is in the steep portion of the oxygen-hemoglobin dissociation curve and increased ${\dot{\text{V}}}_{E}$ could have pronounced effects on arterial oxygen concentration and may help to preserve muscle ${\dot{\text{V}}\text{O}}_{2}$ despite reduced limb blood flow. However, in normoxia the PO_2_ is in the flatter region of the oxygen-hemoglobin dissociation curve and the same increases in ${\dot{\text{V}}}_{E}$ would be less effective in altering arterial oxygen concentration relative to hypoxia. As a consequence the increased work associated with breathing would result in little/small increases in arterial oxygen concentration and reduce muscle blood flow and thus muscle ${\dot{\text{V}}\text{O}}_{2}$. However, it should be noted that exercise induced arterial hypoxemia has also been reported during different exercise modalities, such as cycling (Powers et al., 1988), whereas the phenomenon whereby ${\dot{\text{V}}\text{O}}_{2}$ attains a plateau below ${\dot{\text{V}}\text{O}}_{2\text{max}}$ has only been reported in highly aerobically trained individuals during CWR running exercise lasting ~2 min. The mechanistic origin(s) for this phenomenon is currently unknown and requires further research.

Despite only one transition to the CWR trial in each condition, due to the large amplitude of the ${\dot{\text{V}}\text{O}}_{2}$ response during this intensity of exercise there is a much greater signal/noise ratio when compared to exercise of a lower intensity (Lamarra et al., 1987). In lesser trained individuals with smaller ${\dot{\text{V}}\text{O}}_{2}$ amplitude, thus smaller signal to noise ratio, Draper et al. (2008) demonstrated that two transitions would at worst (i.e., smallest signal to noise ratio) provide 95% confidence intervals of 1 s. Given that the current study recruited more highly aerobically trained individuals than Draper et al. (2008), thus a greater signal to noise ratio, it would be reasonable to expect 95% confidence intervals of better than 2 s for τ. Furthermore, the current study design was sufficiently sensitive and had adequate power to detect differences in τ between conditions.

In conclusion, the results of the present study demonstrate that highly aerobically trained individuals whom are unable to achieve ${\dot{\text{V}}\text{O}}_{2\text{max}}$ during an exhaustive CWR run lasting ~2 min, are able to achieve a hypoxia reduced ${\dot{\text{V}}\text{O}}_{2\text{max}}$ despite exhibiting slower ${\dot{\text{V}}\text{O}}_{2}$ kinetics. These data further support the notion that ${\dot{\text{V}}\text{O}}_{2\text{max}}$ is an important determinant of the ${\dot{\text{V}}\text{O}}_{2\text{max}}$ that can be achieved during a short duration exhaustive CWR run. The present data demonstrate that ventilatory differences are unable to explain the inability to attain ${\dot{\text{V}}\text{O}}_{2\text{max}}$ during normoxic CWR trials. Future research should explore the possibility of an O_2_ delivery or blood perfusion limitation during this type of exercise in highly aerobically trained runners. Future research should also consider utilizing an experimental condition in normoxia which uses gradient on the treadmill (or weighted vest) instead of hypoxia to slow the running speed down and induce task failure in ~2 min. This would aid in deciphering the novel finding of this study.

## Ethics statement

The study was approved by University of Gloucestershire Ethics Committee. All participants were provided with verbal and written information that detailed the rationale of the study, the test procedures, and any risks and benefits of participation. Participants were informed of their right to withdraw from the study at any time without penalty. All participants provided written informed consent detailing that they were willing to take part.

## Author contributions

MB, CP, SD, JC, and CC were involved in conceptual design, data collection, interpretation, and manuscript preparation. All authors approve the submission of this work and agree to be accountable for all aspects of the work.

### Conflict of interest statement

The authors declare that the research was conducted in the absence of any commercial or financial relationships that could be construed as a potential conflict of interest. The reviewer NT and handling Editor declared their shared affiliation, and the handling Editor states that the process nevertheless met the standards of a fair and objective review.
